# Soil organic matter and water content affect the community characteristics of arbuscular mycorrhizal fungi in Helan mountain, an arid desert grassland area in China

**DOI:** 10.3389/fmicb.2024.1377763

**Published:** 2024-06-19

**Authors:** Leilei Shao, Peixuan Yan, Siqi Ye, Hao Bai, Rui Zhang, Guangyao Shi, Yang Hu, Danbo Pang, Xiang Niu, Xilu Ni

**Affiliations:** ^1^Breeding Base for State Key Laboratory of Land Degradation and Ecological Restoration in Northwestern China, School of Ecology and Environment, Ningxia University, Yinchuan, China; ^2^Ningxia Yinchuan Urban Ecosystem Research Station, National Forestry and Grassland Administration, Yinchuan, China; ^3^School of Life Sciences, Southwest University, Chongqing, China; ^4^College of Forestry and Prataculture, Ningxia University, Yinchuan, China; ^5^Ecology and Nature Conservation Institute, Chinese Academy of Forestry, Beijing, China

**Keywords:** mountain ecosystems, AMF community, altitude, soil physicochemical properties, arid desert grassland area

## Abstract

**Introduction:**

Arbuscular mycorrhizal fungi (AMF) are vital in terrestrial ecosystems. However, the community structure characteristics and influencing factors of AMF in the forest ecosystems of arid desert grassland areas require further investigation.

**Methods:**

Therefore, we employed high-throughput sequencing technology to analyze the soil AMF community characteristics at different elevations in the Helan mountains.

**Results:**

The results revealed that significant differences (*P* < 0.05) were observed in the soil physicochemical properties among different elevations, and these properties exhibited distinct trends with increasing elevation. Through high-throughput sequencing, we identified 986 operational taxonomic units (OTUs) belonging to 1 phylum, 4 classes, 6 orders, 12 families, 14 genera, and 114 species. The dominant genus was Glomus. Furthermore, significant differences (*P* < 0.05) were observed in the α-diversity of the soil AMF community across different elevations. Person correlation analysis, redundancy analysis (RDA), and Monte Carlo tests demonstrated significant correlations between the diversity and abundance of AMF communities with soil organic matter (OM) (*P* < 0.01) and soil water content (WC) (*P* < 0.05).

**Discussion:**

This study provides insights into the structural characteristics of soil AMF communities at various altitudes on the eastern slope of Helan mountain and their relationships with soil physicochemical properties. The findings contribute to our understanding of the distribution pattern of soil AMF and its associations with environmental factors in the Helan mountains, as well as the stability of forest ecosystems in arid desert grassland areas.

## 1 Introduction

Mycorrhiza is a symbiotic association formed between the mycelium of fungi and the root system of higher plants. Symbiotic fungi expand the absorptive capacity of plant roots by obtaining nutrients for their growth and development from the plant. Simultaneously, they enhance the plant’s ability to acquire water and nutrients and facilitate interactions between the plant and other soil microorganisms (Tedersoo et al., 2020). Mycorrhizal fungi are important components of natural ecosystems and have a significant impact on plant community composition and ecosystem function (Koide and Mosse, 2004). About 320,000–340,000 plants can form mycorrhizae, with the most widespread distribution of arbuscular mycorrhizal fungi (AMF), which account for about 72% of mycorrhizal fungi (Genre et al., 2020). Moreover, in addition to nutrient uptake, AMF contributes to enhancing plant resistance to environmental and biological stresses, thereby improving plant disease and stress tolerance (Hashem et al., 2021; Dowarah et al., 2022). Several studies comparing different ecosystems have demonstrated a greater reliance on AMF for plant survival in semi-arid habitats (Li and Zhao, 2005). Other studies have shown that the diversity and abundance of AMF communities change along environmental gradients, mainly due to the influence of different external environmental factors such as altitude, soil physicochemical properties, and climate (Bonfim et al., 2016; Prudent et al., 2020). Particularly, factors such as soil moisture, organic matter (OM), and the content of nitrogen and phosphorus elements have a significant impact on the diversity and abundance of AMF communities (Gomoryova et al., 2013; Egan et al., 2017).

Mountain ecosystems serve as natural experimental areas for studying the biodiversity of organisms along environmental gradients, including continuous temperature changes (Barcenas-Moreno et al., 2009), soil physicochemical properties, and vegetation composition (Wang et al., 2022), providing valuable research opportunities. Previous research results have shown that the abundance and diversity of microorganisms exhibit different trends with changes in elevation gradient. Fungi in Andean Yungas forests (Wang et al., 2015) and bacteria near Gunnison County, Colorado (Bryant et al., 2008) exhibited a decrease in both diversity and abundance with increasing elevation. The relative abundance of *Glomus* in the Guangxi hills of China increased with elevation (Yu et al., 2023). In the inter-root soils of the Taibai Mountains in China, microbial α-diversity initially increased and then decreased with elevation (Ren et al., 2021). In the arid valley of Panzhihua, there was a trend of decreasing and then increasing microbial community richness with elevation (Zhang R. J. et al., 2022), and it has also been demonstrated that the diversity of the AMF community is not correlated with elevation (Shen et al., 2014). These observations can be attributed to the fact that environmental conditions vary across regions and elevations, creating complex circumstances for microbial survival and distribution. Additionally, slope orientation is an important influencing factor, as there are significant differences in vegetation types, soil physicochemical properties, light exposure, and temperature between shady and sunny slopes, which may have a significant impact on microbial abundance and diversity (Guo et al., 2022).

As an important ecological barrier in northwest China, the Helan mountains exhibit significant vertical vegetation zonation and a complete vegetation spectrum. Along the altitude gradient, vegetation types transition from desert grasslands to shrublands, sparse forests, subalpine coniferous forests, and finally to alpine shrub meadows (Jiang et al., 2000). The diversity and abundance of AMF are closely related to these different vegetation types (Uhlmann et al., 2004; Dassen et al., 2017), and vegetation diversity influences the community structure of AMF (Prommer et al., 2020). At the same time, AMF plays an important role in maintaining the stability of plant communities. There are relatively limited studies on the diversity and distribution of AMF communities and the main factors affecting them in the Helan mountains. In this study, we focused on the eastern slope of the Helan mountains as the study area and used high-throughput sequencing technology to investigate the characteristics of AMF communities in the rhizosphere soil of plants at different altitudes on the sunny slope of the eastern slope. We also analyzed the correlation between various soil physicochemical indicators and AMF community characteristics. This study aimed to reveal the distribution patterns of AMF communities and their influencing factors, offering valuable reference for the maintenance of mountainous ecosystem stability.

## 2 Materials and methods

### 2.1 Overview of the sampling area

Helan mountain is situated between the Alashan Plateau and the Yinchuan Plain, spanning coordinates 38°27′-39°30′N and 105°41′-106°41′E. The highest altitude of the sampling point reaches 2,580 m. The Helan mountains have a typical continental climate, with an average annual temperature of −1 to 8°C, an average annual precipitation of 200–400 mm, and a large average annual evaporation of more than 2,000 mm. The sampling area is situated on the sunny slope of the eastern side of the Helan mountains. Common plant species in this region include *Roegneria alashanica*, *Aristida adscensionis*, *Stipa breviflora*, *Prunus mongolica*, *Ulmus glaucescens, Picea crassifolia*, and so on. The soil is mostly sandy loam.

### 2.2 Experimental design

The sample plots were established at altitudes ranging from 1,349 to 2,580 m, covering typical plant communities on the eastern slope of the Helan mountains, including grassland (GR), shrubs (SH), *U. glaucescens* forests (UL), *Pinus tabuliformis* forests (PF), coniferous and broad-leaved mixed forests (CB), *P. crassifolia* forests (PI), and subalpine meadow (SU), as shown in Table 1. Within each sampling area, three 20 m × 20 m quadrats were established. The five-point sampling method was used to collect plant inter-root soil from 0 to 20 cm depth with a 4 cm diameter soil sampler, which was mixed and sealed in a sterilized bag, transferred to the laboratory in a refrigerated box, and a part of the soil was air-dried and stored at room temperature, while the other part was stored in a refrigerator at −80°C to be preserved and used for soil microbial sequencing (Schleuss and Muller, 2001).

**TABLE 1:** Basic information of sample plots.

Plot	Vegetation type	ALT (m)
GR	Grassland	1,349
		1,349
		1,349
SH	Shrubs	1,670
		1,676
		1,716
UL	*Ulmus glaucescens* forests	2,012
		2,022
		2,043
PF	*Pinus tabuliformis* forests	2,277
		2,283
		2,305
CB	Coniferous and broad-leaved mixed forests	2,336
		2,340
		2,344
PI	*Picea crassifolia* forests	2,401
		2,510
		2,570
SU	Subalpine meadow	2,580
		2,580
		2,580

### 2.3 Experimental measurement methods

#### 2.3.1 Determination of soil physical and chemical properties

Soil pH was determined using the potentiometric method, with a soil-to-liquid ratio of 1:2.5. Soil water content (WC) was determined using the drying method. Soil-available phosphorus (AP) was measured using a 0.5 mol⋅ L^–1^ NaHCO_3_ solution. Soil total phosphorus (TP) was analyzed using the perchloric acid-sulfuric acid solubilization method, followed by the molybdenum antimony antimicrobial colorimetric method. Soil OM was quantified using the potassium dichromate oxidation-external heating method. Total nitrogen (TN) in the soil was determined using the Kjeldahl method. Soil available nitrogen (AN) was measured using the alkaline dissolution-diffusion method. Soil-available calcium (AK) was analyzed using the ammonium acetate-flame photometer method (Lv and Li, 2010).

#### 2.3.2 Determination of soil AMF community diversity

Arbuscular mycorrhizal fungi community diversity was determined by Majorbio Bio-Pharm Technology Co., Ltd. (Shanghai, China). Soil AMF community DNA was extracted by MP kit using AMV4-5NF/AMDGR primers with primer sequences of 5′-AAGCTCGTAGTTGAATTTCG-3′, 5′-CCCAACTATCCCTATTAATCAT-3′, and PCR for 18S rRNA gene amplification. The TransGen AP221-02 kit containing TransStart Fastpfu DNA polymerase was used for the PCR reaction. (1) PCR products from the same sample were mixed and detected by 2% agarose gel electrophoresis, (2) PCR products were recovered by cutting the gel using the AxyPrepDNA Gel Recovery Kit, (3) Tris_HCl elution; 2% agarose electrophoresis was detected. (4) Sodium hydroxide denaturation to generate single-stranded DNA fragments (Kowalchuk et al., 2002). DNA bipartite sequencing was performed on the Illumina MiSeq PE300 platform (Illumina, San Diego, CA, USA). Using UPARSE (Edgar, 2013) software (version 11),^1^ OTUs were classified based on a 97% sequence similarity threshold. The representative sequences of OTUs with 97% similarity were taxonomically analyzed using the RDP classifier Bayesian algorithm. Finally, the taxonomic information was obtained from the maarjam20220506/AM species classification database.

### 2.4 Data analysis

The data were organized using Excel 2021, and SPSS 26 was utilized for data processing. To analyze the differences in soil physicochemical properties among vegetation zones (*P* < 0.05), one-way ANOVA and Tukey’s honest Significant Difference test were employed. Pearson’s correlation analysis (PCA) with a two-tailed significance test was used to explore the correlations between different variables. Non-metric multidimensional scaling analysis (NMDS) and analysis of similarity test (ANOSIM) were selected to examine the differences among AMF communities. Bar scale plots were generated using Origin 2022. The computation platform provided by Shanghai Meiji Biomedical Technology Co. was used for the analysis and mapping of soil AMF community diversity and its correlation with environmental factors.

Soil AMF community α-diversity analysis was conducted using the Shannon-Wiener diversity index (H), Simpson dominance index (D), Chao1 index (S_*obs*_), and ACE index (S_*ACE*_).

① Shannon-Wiener index (H):


$$H=-\sum_{i=1}^{S_{o\,b\,s}}\frac{n_{i}}{N}\,l\,n\,\frac{n_{i}}{N}$$

② Simpson dominance index (D):


$$\text{D}=-\frac{\sum_{\text{i}=1}^{{\text{S}}_{\text{obs}}}{\text{n}}_{\text{i}}\,\left({\text{n}}_{\text{i}}-1\right)}{N\,\left(N-1\right)}$$

Where: S_*obs*_ is the number of OTUs actually observed; n_*i*_ is the number of sequences contained in the *i*-th OTU and N is the number of all sequences.

③ Chao1 index (S_*obs*_):


$$S_{c\,h\,a\,o\,1}=S_{o\,b\,s}+\frac{F_{1}\,\left(F_{1}-1\right)}{2\,\left(F_{2}+1\right)}$$

Where: S_*chao1*_ is the estimated number of OTUs; F_1_ is the number of OTUs containing only one sequence; and F_2_ is the number of OTUs containing only two sequences.

④ ACE index (S_*ACE*_):


$$S_{A\,C\,E}=S_{a\,b\,u\,n\,d}+\frac{S_{r\,a\,r\,e}}{C_{A\,C\,E}}+\frac{F_{1}}{C_{A\,C\,E}}\,\gamma_{A\,C\,E}^{2}$$

Where, $\gamma_{A\,C\,E}^{2}=\max\left[\frac{S_{r\,a\,r\,e}\,\sum_{i=1}^{10}i\,\left(i-1\right)\,F_{i}}{C_{A\,C\,E}\,N_{r\,a\,r\,e}\,\left(N_{r\,a\,r\,e}-1\right)}-1,0\right]$


$$C_{A\,C\,E}=1-\frac{F_{1}}{N_{r\,a\,r\,e}}$$

$$N_{r\,a\,r\,e}=\sum_{i=1}^{10}i\,F_{i}$$

S_*rare*_ is the number of OTUs with no more than “abund”” sequences; S_*abund*_ is the number of OTUs with more than “abund” sequences; abund is the threshold for “dominance” OTUs. abund is the threshold of “dominance” OTUs, and the default is 10.

## 3 Results

### 3.1 Physical and chemical properties of soils at different altitudes

The physicochemical properties of soils at different altitudes on the eastern slope of Helan mountain exhibited significant variations (*P* < 0.05), as presented in Table 2. WC and TP displayed an increasing trend with altitude, reaching their highest values in the high-altitude area (13.07% and 161.84 mg/kg, respectively). On the other hand, OM, TN, AN, AP, and AK demonstrated a pattern of initially increasing and then decreasing along the altitude gradient. The middle altitude area had the highest levels of OM, AN, and TN (9.25%, 137.26 mg/kg, and 165.77 mg/kg, respectively), while AP reached its peak in the high-altitude area (12.43 mg/kg). AK, on the other hand, exhibited its highest concentration in the low-altitude region (49.83 mg/kg). Soil pH decreased with altitude, ranging from 7.95 to 8.13, with minor fluctuations, indicating a slightly alkaline nature of the soil on the eastern slope of Helan mountain. In summary, the nutrient content of soils on the eastern slope of Helan mountain increased with altitude, in agreement with the findings of previous studies.

**TABLE 2:** Physicochemical properties of soils of different vegetation types.

PLOT	WC/%	pH	TN/(mg/kg)	AN/(mg/kg)	TP/(mg/kg)	AP/(mg/kg)	AK/(mg/kg)	OM/%
GR	2.01 ± 0.28c	8.13 ± 0.01a	44.16 ± 15.74d	30.39 ± 4.56c	76.71 ± 4.03d	6.86 ± 0.33c	23.87 ± 7.12e	2.21 ± 0.32e
SH	1.86 ± 0.26c	8.10 ± 0.09a	64.71 ± 16.87cd	39.29 ± 18.96c	146.32 ± 5.82b	6.12 ± 1.55c	49.83 ± 7.94a	3.82 ± 0.74de
UL	3.18 ± 0.45c	8.06 ± 0.07ab	133.83 ± 11.23b	82.36 ± 23.03b	153.58 ± 9.91ab	10.26 ± 1.15b	42.27 ± 1.03abc	6.58 ± 0.92bc
PF	6.70 ± 0.77b	8.09 ± 0.10a	172.73 ± 4.95a	137.26 ± 6.64a	140.65 ± 8.67b	8.96 ± 0.63b	36.80 ± 5.37bcd	9.25 ± 1.13a
CB	7.76 ± 1.14b	7.95 ± 0.05b	74.29 ± 11.26c	45.21 ± 11.01c	68.02 ± 3.55d	5.62 ± 1.64c	33.67 ± 4.52cde	7.00 ± 1.77bc
PI	13.07 ± 1.01a	7.96 ± 0.05b	56.56 ± 13.31cd	40.15 ± 11.70c	161.84 ± 4.97a	12.43 ± 0.080a	30.67 ± 9.22de	7.68 ± 0.68ab
SU	2.40 ± 0.47c	8.09 ± 0.04a	165.77 ± 15.71a	88.71 ± 0.61b	111.04 ± 15.49c	6.76 ± 1.04c	47.80 ± 1.80ab	5.24 ± 1.11cd

Different lowercase letters in the same column indicate significant differences (*P* < 0.05). Values are means ± SE (*n* = 3).

### 3.2 α-Diversity of soil AMF communities at different altitude gradients of Helan mountain

**FIGURE 1:**
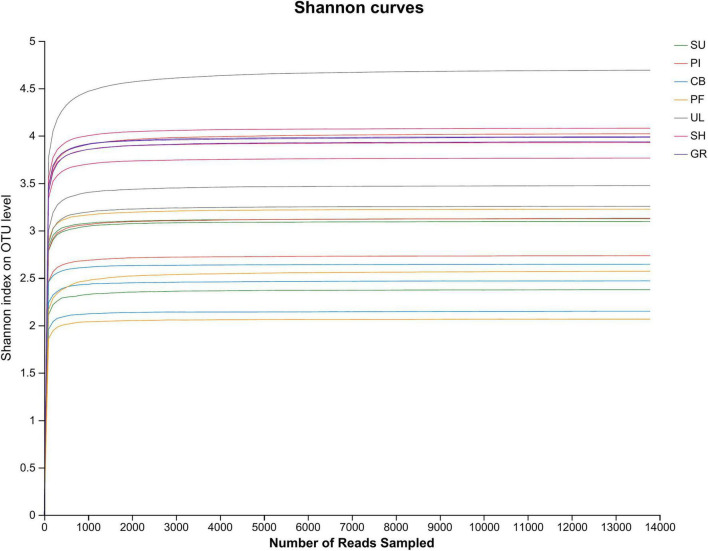
Dilution curve of soil AMF community.

As shown in Figure 1, when the end of the dilution curve of the Shannon index tends to flatten toward flat, it indicates that the amount of sequencing data in the sample is reasonable enough. That is, the biodiversity in the sample is high, and there exists a relative abundance of different species or gene expression levels that can be reasonably analyzed for subsequent data.

Table 3 displays significant differences (*P* < 0.05) in the α-diversity of AMF communities among different vegetation types and altitude gradients. Specifically, the soil AMF community in GR exhibited the highest Shannon index and the lowest Simpson index, while the soil AMF community in CB showed the lowest Shannon index and the highest Simpson index. This indicates that the α-diversity of soil AMF communities was highest in desert grasslands and lowest in mixed coniferous forests. Regarding the ACE index and Chao1 index, UL had the highest values, whereas CB had the lowest. No significant differences were observed among vegetation types at different altitudes.

**TABLE 3:** Soil AMF diversity index for different vegetation types.

PLOT	Shannon	Simpson	ACE	Chao1	Coverage
GR	3.97 ± 0.03a	0.04 ± 0.00c	185.84 ± 10.37a	185.99 ± 11.62a	0.9988 ± 0.0004a
SH	3.92 ± 0.16a	0.04 ± 0.01c	169.97 ± 26.11a	167.57 ± 24.84a	0.9990 ± 0.0005a
UL	3.81 ± 0.77a	0.06 ± 0.03c	309.19 ± 260.56a	303.35 ± 252.03a	0.9972 ± 0.0031a
PF	2.62 ± 0.58b	0.17 ± 0.08ab	162.06 ± 101.67a	164.08 ± 97.13a	0.9984 ± 0.0011a
CB	2.42 ± 0.25b	0.17 ± 0.05a	96.75 ± 12.05a	93.43 ± 14.35a	0.9991 ± 0.0001a
PI	3.29 ± 0.66ab	0.08 ± 0.05bc	218.43 ± 115.80a	215.64 ± 103.87a	0.9971 ± 0.0020a
SU	2.86 ± 0.42b	0.13 ± 0.06abc	133.69 ± 43.99a	141.39 ± 25.26a	0.9986 ± 0.0006a

Observed α-diversity results are based on the number of OTUs in each group. Different lowercase letters indicate significant differences between treatments (*P* < 0.05). Values are means ± standard errors (SE; *n* = 3).

In summary, the α-diversity of soil AMF communities on the eastern slope of the Helan mountains exhibited significant variations among different vegetation types. The overall trend indicated a decrease followed by an increase in altitude. Overall, gray elm forests showed the highest alpha diversity among soil AMF communities, while mixed coniferous and broadleaf forests displayed the lowest diversity.

### 3.3 Taxonomic composition and distribution of soil AMF at different altitudes of Helan mountain

A total of 986 OTUs of AMF were identified through high-throughput sequencing in this study. These OTUs belonged to 1 phylum, 4 classes, 6 orders, 12 families, 14 genera, and 114 species.

**FIGURE 2:**
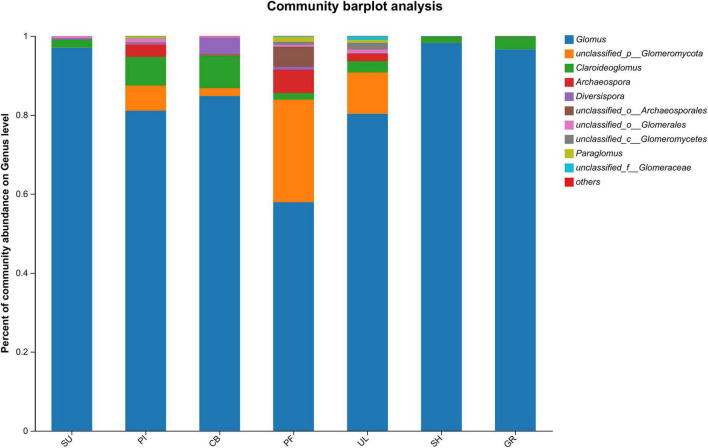
Community composition of soil AMF.

**FIGURE 3:**
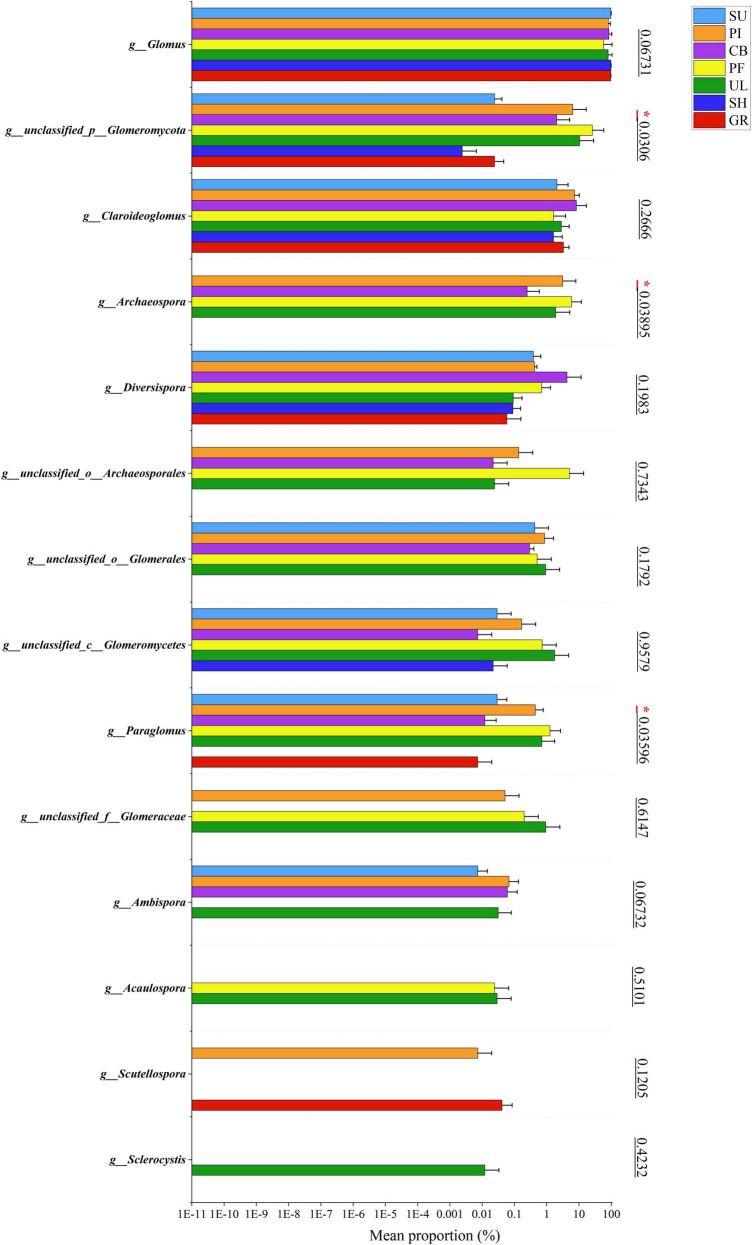
Significance test for difference in AMF species at different altitude. The symbol “*” heatmap between soil factors represents significant correlations at the 0.05 level.

Figure 2 illustrates significant variations in the relative abundance of soil AMF at the genus level among samples at different altitudes. Among all the samples, *Glomus* exhibited the highest relative abundance, with an average percentage of 85.13%. The highest relative abundance (98.27%) was observed in sample SH at a low altitude, indicating that *Glomus* was the dominant species in the soil AMF community on the eastern slope of the Helan mountains. In addition, the 2nd to 5th relative abundances were unclassified__p__*Glomeromycota*, *Claroideoglomus*, *Archaeospora*, and unclassified__o_*Archaeosporales* in the order of relative abundance. As shown in Figure 3, further analysis of the species differences at the genus level across different samples revealed that *Glomus*, unclassified_p_*Glomeromycota*, *Claroideoglomus*, and *Diversispor*a were present in all altitudinal gradient samples, indicating their wide distribution on the eastern slopes of Mount Helan. *Sclerocystis* was found exclusively in sample UL, while unclassified_p_*Glomeromycot*a, *Archaeospora*, and *Paraglomus* exhibited significant differences (*P* < 0.05) among samples.

**FIGURE 4:**
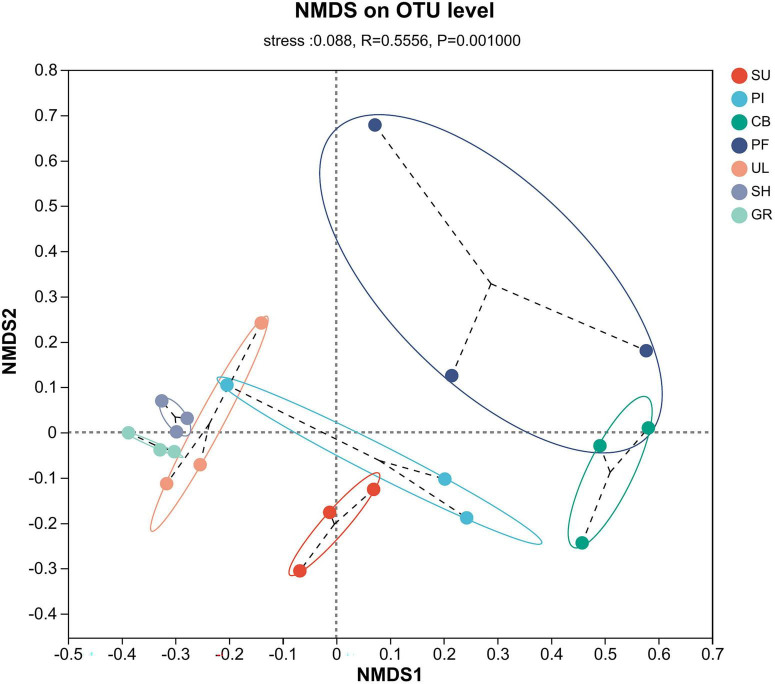
Non-metric multidimensional scaling analysis of β-diversity of soil AMF communities.

Comparative analysis of AMF community species diversity among different samples was conducted at the OTU level. The results of NMDS analysis, utilizing the Bray–Curtis distance algorithm, and the ANOSIM test revealed significant differences in the community structure of AMF across the samples (*R* = 0.5556, *P* = 0.001). As depicted in Figure 4, the samples exhibited distinct clustering patterns in a two-dimensional space. The PF sample points at the middle altitude appeared more scattered, indicating lower species similarity among those samples. Conversely, the SH and GR sample points at the low altitude were more closely grouped, suggesting higher species similarity. Notably, the NMDS1 axis clearly distinguished the AMF communities between high and low altitudes. The greatest species differences were observed between the SH and GR communities at low altitudes and the PF and CB communities at medium altitudes.

**FIGURE 5:**
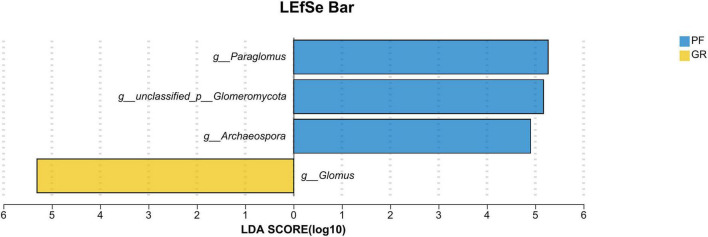
LEfSe bar analysis of GR vs. PF.

To further investigate the differences between the PF and GR samples, LEfse analysis (Figure 5) was performed. The analysis revealed that the abundance of *Glomus* was significantly higher in GR compared to PF. Conversely, the abundance of unclassified__p__*Glomeromycota*, *Archaeospora*, and *Paraglomus* was significantly higher in PF compared to GR.

### 3.4 Correlation analysis between α-diversity of soil AMF communities and soil physicochemical properties at different altitude gradients of Helan mountain

The results of Pearson correlation analysis (Table 4) revealed significant associations between soil AMF community α-diversity indices and various soil factors. The Shannon index exhibited a highly significant positive correlation with soil OM and a significant positive correlation with WC. Conversely, the Simpson index showed a highly significant negative correlation with OM and a significant negative correlation with WC. The ACE index was significantly positively correlated with ALT and OM, while the Chao1 index demonstrated a highly significant positive correlation with WC and OM, as well as a significant positive correlation with ALT.

**TABLE 4:** Person correlation analysis between soil physical and chemical properties and soil AMF community α-diversity.

	ALT	Shannon	Simpson	ACE	Chao1
ALT	1	0.3	−0.291	0.449*	0.516*
pH	−0.467*	−0.199	0.211	−0.422	−0.348
WC	0.577**	0.518*	−0.522*	0.362	0.570**
OM	0.699**	0.678**	−0.663**	0.467*	0.673**
TN	502*	0.202	−0.173	0.263	0.244
AN	0.419	0.219	−0.187	0.19	0.184
TP	0.215	0.296	−0.28	0.062	0.264
AP	0.301	0.327	−0.313	0.118	0.329
AK	0.201	−0.22	0.228	−0.158	−0.203

*Significant at the 0.05 level (two-tailed).

**Significant correlation at the 0.01 level (two-tailed).

**FIGURE 6:**
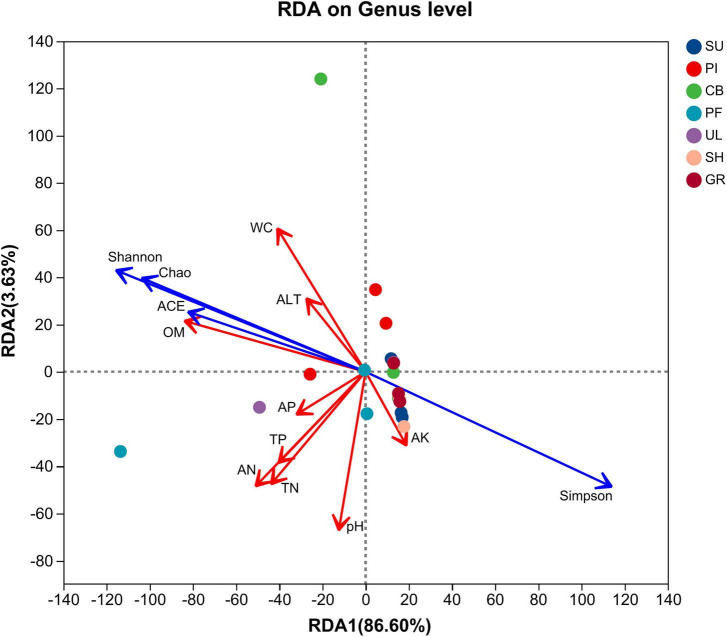
Redundancy analysis of soil environmental factors and diversity coefficients of AMF communities.

To explore the relationship between soil AMF community α-diversity and environmental factors, redundancy analysis (RDA) analysis was conducted (Figure 6). The first and second ordination axes explained 86.60% and 3.63% of the variance in AMF community composition, respectively. The Shannon, ACE, and Chao indices exhibited positive correlations with AP, OM, ALT, WC, total TN, AN, and TP, while showing negative correlations with AK and pH. Simpson’s index displayed the opposite pattern. The results of the Monte Carlo significance test indicated that OM (*r*^2^ = 0.3926, *P* = 0.003) and WC (*r*^2^ = 0.2715, *P* = 0.049) were the primary environmental factors influencing the α-diversity of AMF communities.

**FIGURE 7:**
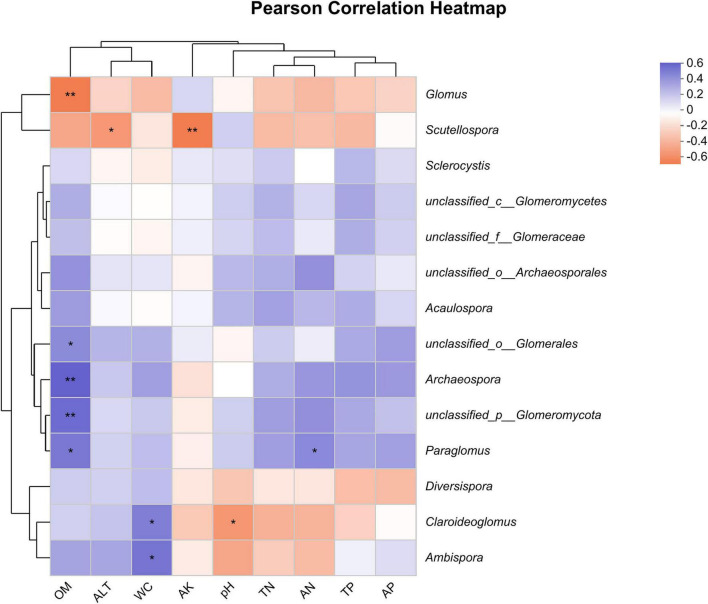
Person correlation heatmap between soil factors and AMF community. The symbols * and ** represent significant correlations at 0.05 and 0.01 levels, respectively.

The results of the heatmap plot of environmental factors and soil AMF genus level showed (Figure 7) that different environmental factors had different degrees of influence on soil AMF genera. Among them, soil OM showed a highly significant positive correlation with the genera unclassified_ p__*Glomeromycota* and *Archaeospora*, a significant positive correlation with the genera *Paraglomus* and unclassified_o_*Glomerales*, and a highly significant negative correlation with *Glomus*. WC was positively correlated with *Ambispora* and *Claroideoglomus*, AN was positively correlated with *Paraglomus*, *Scutellospora* was most affected by ALT and AK, and *Claroideoglomus* was most affected by WC and pH. In addition, the effects of environmental factors on unclassified__o_*Archaeosporales*, *Acaulospora*, *Sclerocysti*s, unclassified__c__*Glomeromycetes*, unclassified__f__*Glomeraceae*, and *Diversispora* had no significant effect.

## 4 Discussion

### 4.1 Characterization of AMF community structure in soils at different altitudes

Consistent with the results of many studies (Zhang M. G. et al., 2022), *Glomus*, as the most common genus of AMF, is widely distributed in the natural environment (Schwarzott et al., 2001). In our study, *Glomus* also emerged as the dominant genus in the AMF community of the soil on the east slope of Helan mountain. This can be attributed to *Glomus’* exceptional reproductive capacity and its ability to establish mycorrhizal associations rapidly with host plants. These symbiotic relationships enhance the nutrient and water absorption capabilities of host plants, promote plant growth efficiency, and improve stress resistance (Beimforde et al., 2014; Zhao et al., 2017). Consequently, *Glomus* contributes to the stability of plant communities and indirectly enriches soil biodiversity. Precipitation enhances vegetation photosynthesis efficiency, leading to increased biomass in host plants and providing more nutrients for AMF (Manzoni et al., 2012). However, as altitude rises, precipitation and soil WC increase and plant roots become less reliant on AMF for water uptake. Furthermore, the decrease in temperature at higher altitudes hinders the growth and development of AMF (Yang et al., 2022). Since *Glomus* encompasses a greater number of species, the adverse effects of environmental changes associated with increased altitude are more pronounced for this genus compared to others (Zhang M. G. et al., 2022).

Significant differences in soil AMF community α-diversity were observed among different altitudes. Consistent with previous studies (Guo et al., 2020), the α-diversity of soil AMF communities on the eastern slopes of the Helan mountains exhibited a positive correlation with ALT. This suggests that altitude and topography influence variations in soil properties and vegetation types, which, in turn, impact the AMF and mycorrhizal environments. The influence of host plants on soil AMF community α-diversity is substantial (Li et al., 2014; Davison et al., 2015). The Helan mountains exhibit distinct vertical distribution patterns in vegetation zones, giving rise to significant differences in soil AMF across these zones (Li et al., 2015). Research has established a direct correlation between soil AMF community diversity and plant community diversity and stability (Stover et al., 2018; Zhao et al., 2022). The region with the highest soil AMF diversity on the eastern slopes of the Helan mountains is situated in the lower altitude GR. Although the vegetation diversity and richness in the GR are not particularly high, the presence of dense shrubs and grasses, coupled with lower altitudes and favorable temperatures, fosters increased wildlife activity and appropriate disturbances. These factors accelerate material cycling, and energy flow, and increase the diversity of soil AMF. UL located at mid-altitude showed the highest AMF abundance among the seven experimental sample sites. This may be because the diversity and richness of vegetation in this region is significantly higher than that at lower altitudes, providing a wider range of hosting options for AMF. The PI also showed relatively higher abundance and diversity at higher altitudes. This may be because the PI is a relatively mature forest area with a more stable plant community. It may also be that in arid and semi-arid regions, mycorrhizal hyphae are more important to plants due to low soil WC (Bahadur et al., 2019). The increase in soil WC at high altitudes promotes the reproduction and growth of plant-AMF symbionts.

### 4.2 Main factors influencing soil AMF community structure

The composition and distribution of AMF communities on the eastern slopes of the Helan mountains are significantly influenced by differences in soil physical and chemical properties, as indicated by the findings of this study. Correlation analysis revealed that soil OM and WC exerted a stronger impact on the α-diversity of AMF communities compared to other soil physical and chemical properties. This can be attributed to WC promoting the reproductive development of AMF and influencing the selection and colonization of AMF by host plants (Shukla et al., 2013). OM serves as a carbon source necessary for microbial reproduction and positively affects AMF spore density and colonization, thereby enhancing AMF reproductive capacity to a certain extent (Bending et al., 2002; Shao et al., 2019; Sadeghi et al., 2023). Interestingly, the relative abundance of *Glomus*, the dominant genus in the study area, exhibited a pattern of decreasing and then increasing along different altitudinal gradients, which contradicted the trend observed for OM. This suggests that factors such as temperature at lower altitudes and soil moisture content at higher altitudes positively promote the growth and reproduction of *Glomus* as the dominant genus. It indicates that *Glomus* is not solely reliant on soil OM and possesses adaptability to nutrient-poor soil environments. Alternatively, the growth of *Glomus* may be limited by chemical inhibition or nutrient competition from plants (Singh et al., 2007).

This study conducted Person correlation analysis and found a highly significant positive correlation between altitude and soil OM, demonstrating the significant influence of altitude on the α-diversity of AMF communities. As a bridge for nutrient conversion between plant roots and the soil environment, the diversity of AMF is inevitably affected by inter-root soil OM (Zinger et al., 2011). Excessively high or low nutrient levels can inhibit AMF colonization and growth, with soil nitrogen and OM content playing a key role in determining the abundance and diversity of AMF (Xiao et al., 2018). Although soil nitrogen content was not the primary factor influencing AMF diversity and richness in this study, TN showed a significant positive correlation with altitude in Person’s correlation analysis, indicating that altitude indirectly influenced the α-diversity of AMF communities by affecting soil nitrogen content. Contrary to the results of previous studies by Shen et al. (2013), Guo and Gong (2014), and Li et al. (2022), this study found that pH was not the main factor influencing the AMF community structure on the east slope of Helan mountain. This discrepancy may be attributed to the limited range of soil pH variation in the samples of the present experiment.

Among the seven sample sites in this study, the middle altitude area, represented by the UL, exhibited medium levels of soil physicochemical indices, and the richness and diversity of the AMF community were relatively high across all sample sites. In the high-altitude region, represented by the PI, all soil physicochemical properties were at the upper range among the experimental sample plots, but the AMF community diversity was not high. In the low-altitude regions (SH and GR), the levels of soil physical and chemical properties were relatively lower, yet the AMF community diversity was high. This may be attributed to the fact that low and middle altitudes have more suitable temperatures that positively impact AMF compared to soil nutrients and moisture. Additionally, the timing of sampling may have influenced the results (Liu et al., 2021).

## 5 Conclusion

The present study investigated the diversity and abundance of soil AMF along altitudinal gradients on the eastern slopes of the Helan mountains. The findings are as follows:

1. Significant differences in soil physicochemical properties were observed at different altitudes. Soil moisture increased with altitude, while nutrient levels showed an initial increase followed by a decrease.
2. High-throughput sequencing detected a total of 986 OTUs belonging to 1 phylum, 4 classes, 6 orders, 12 families, 14 genera, and 114 species. The dominant genus was *Glomus*, accounting for an average proportion of 85.13%. The α-diversity of soil AMF communities exhibited significant differences across different altitudes, generally showing an increasing trend followed by a decrease.
3. Person correlation analysis and RDA analysis revealed that soil OM and WC were the primary factors influencing the diversity and abundance of soil AMF communities on the eastern slopes of the Helan mountains. Nitrogen, phosphorus, and quick-acting potassium were identified as secondary factors affecting the diversity and abundance of soil AMF communities.

As the study progresses, a deeper understanding of soil microbial diversity, distribution patterns, and the underlying mechanisms in the Helan mountains will be revealed. This knowledge will enable better utilization of the ecological functions of soil microbes and ultimately contribute to environmental improvement and ecological restoration.

## Data availability statement

The datasets presented in this study can be found in online repositories. The names of the repository/repositories and accession number(s) can be found below: NCBI – https://www.ncbi.nlm.nih.gov/sra/PRJNA1109613.

## Author contributions

LS: Data curation, Formal analysis, Methodology, Software, Writing – original draft, Writing – review & editing. PY: Data curation, Methodology, Software, Writing – review & editing. SY: Investigation, Writing – review & editing. HB: Investigation, Writing – review & editing. RZ: Investigation, Writing – review & editing. GS: Investigation, Writing – review & editing. YH: Investigation, Writing – review & editing. DP: Investigation, Writing – review & editing. XLN: Funding acquisition, Project administration, Supervision, Writing – review & editing. XN: Funding acquisition, Project administration, Supervision, Writing – review & editing.

## Footnotes

1
http://drive5.com/uparse/
